# The complete mitochondrial genome of *Cyamophila willieti* (Wu) (Hemiptera: Psyllidae)

**DOI:** 10.1080/23802359.2019.1681922

**Published:** 2019-10-24

**Authors:** Xianmei Song, Yunchuan He, Xinpu Wang, Xin Gu

**Affiliations:** School of Agriculture, Ningxia University, Yinchuan, Ningxia, People's Republic of China

**Keywords:** *Cyamophila willieti*, mitochondrial genome, phylogeny

## Abstract

*Cyamophila willieti* (Hemiptera: Psyllidae) is an important insect pest of *Sophora japonica*. In this study, the complete mitochondrial genome of *C. willieti* (GenBank accession number MN364946) was sequenced using Illumina HiSeq X Ten. The mitogenome is 15,809 bp long, and comprises 13 protein-coding genes (PCGs), 2 ribosomal RNA genes (rRNAs), 22 transfer RNA genes (tRNAs), and putative control region (CR). The nucleotide composition of *C. willieti* mitochondrial genome is 37.96% of A, 35.88% of T, 15.98% of C and 10.18% of G. Two rRNAs are located between tRNA-Leu and CR, separated by tRNA-Val. The CR, located between 12 s rRNA and tRNA-Ile, is 844 bp long. The length of 22 tRNAs range from 60 to 70 bp. Phylogenetic analysis showed that *C. willieti* belongs to Psyllinae, genetically close to other four species belonging to the same subfamily. *Cyamophila willieti* mitogenome provides an important data resource for further studies and contributes to our understanding of the phylogeny of this group.

*Cyamophila willieti* belongs to the family Psyllidae of order Hemiptera. There are 1001 known species belonging to 113 genera in China. *Cyamophila willieti* is one of the most primary insect pests of *Sophora japonica* and *S. japonica* var*. endula*. Adults and nymphs of *C. willieti* suck juices on buds, tender tips, leaves, and inflorescences of flowering stage. Their excretion is easy to induce the occurrence of sooty blotch and influence photosynthesis. Then, leaves become shrivelled to dry, petioles are drooping off ahead of time, new shoot growth is slow and inflorescences flowering are inhibited (Yang et al. 2002). Biological characteristics, occurrence regularity, forecast control experiments, relation between major environmental factors, and occurrence of *C. willieti* have been reported (He et al. 2005; Wang et al. 2007; Shen, Chang, Zhang, Han, et al. 2008; Shen, Chang, Zhang, Zhu, et al. 2008; Deng et al. 2014; Yang and Wu 2014). However, the molecular data of *C. willieti* has not been reported. In this paper, the mitochondrial genome of *C. willieti* was sequenced and analyzed, and phylogenetic trees were established to provide important molecular support for the identification of this species, and clarify its phylogenetic status in Hemiptera, which are of great significance in the early warning of pests and prevention of biological invasion.

The sample of *C. willieti* was collected from Yinchuan, Ningxia, China (38°29′59″N, 106°8′9″E) in May 2019, and deposited in the insect herbarium, School of Agriculture, Ningxia University (SANXU, voucher number: HMS201905-01). The complete mitochondrial genome of *C. willieti* (GenBank accession number MN364946) was sequenced using Illumina HiSeq X Ten. Genes were assembled by MITObim v1.9 (Hahn et al. 2013). tRNAs and rRNAs annotations were confirmed and corrected by MITOS at online (Bernt et al. 2013).

This mitogenome is a circular DNA molecule of 15,809 bp in length and contain the typical set of 37 genes, including 13 protein-coding genes (PCGs) (ATP6, ATP8, COI-III, ND1-6, ND4L, and CYTB), 2 ribosomal RNA genes (rRNAs) (12S rRNA and 16S rRNA), 22 transfer RNA genes (tRNAs), and a putative CR. And, the nucleotide composition of *C. willieti* mitochondrial genome is 37.96% of A, 35.88% of T, 15.98% of C, 10.18% of G, 73.84% of A + T content. The start codon ATA was shared with COX2, ATP8, CYTB, ND1, ND2 and ND6; the start codon ATG was shared with COX1, COX3, ATP6 and ND4; ND3 started with codon ATT; ND5 started with codon GTG; and the ND4L started with codon TTG. The conservative stop codon TAA was shared with ATP6, ATP8, COX3, ND1, ND3 and ND6; the stop codon TAG was shared with ND4L; COX1 stop with TA–, and other PCGs end with the single nucleotide T––. TA– and T–– denote that the TAA stop codon is presumed to be completed by the addition of 3′A residues to the mRNA. 16S rRNA and 12S rRNA are located between tRNA-Leu and D-loop, separated by tRNA-Val. The 16S rRNA is 1177 bp and the 12S rRNA is 744 bp in length. The length of 22 tRNAs range from 60 bp (tRNA-Ala and tRNA-His) to 70 bp (tRNA-Lys). The CR, located between 12 s rRNA and tRNA-Ile, is 844 bp in length. This mitogenome has a total of 66 bp overlap sequences and 904 bp intergenic spacer sequences, which are respectively made up of 11 and 8 regions in the range from 1 to 21 bp and 1 to 871 bp. And, the longest overlap and intergenic spacer are respectively located at 16S rRNA and tRNA-Ile.

We selected another 8 Psyllidae species and three outgrous (*Pariaconus pele*, *Paratrioza sinica* and *Trioza urticae*) to reconstruct the phylogenetic tree. The phylogenetic tree was reconstructed using Bayesian 3.2.0 (Ronquist et al. 2012) based on 13 mitochondrial protein-coding genes. The best-fit nucleotide substitution model were selected as ‘GTR + G+I’ using the Akaike Information Criterion (AIC) in jModelTest 0.1.1 (Posada 2008). Phylogenetic analysis showed that *C. willieti* belongs to Psyllinae, genetically close to other four species belonging to the same subfamily (Figure 1), which is consistent with the traditional taxonomic framework. Present study provides an important data resource for further studies of *C. willieti* and contributes to our understanding of the phylogeny of this group.

**Figure 1.**
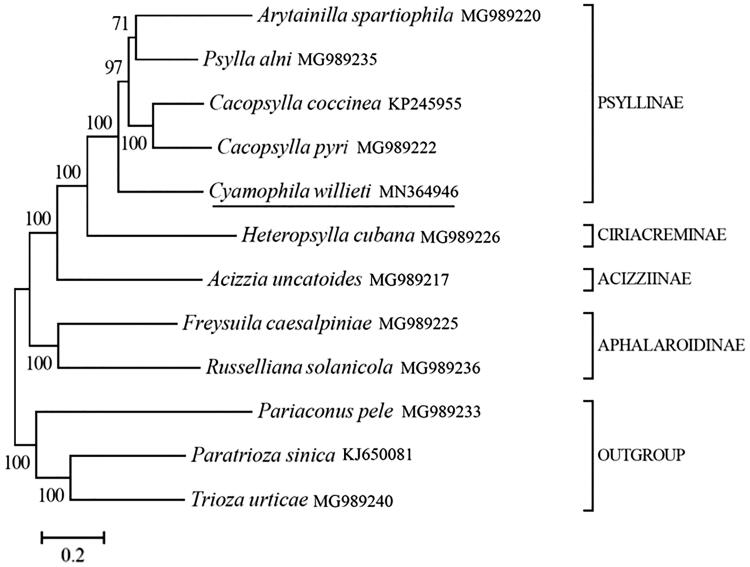
Phylogeny of nine Psyllidae species based on 13 mitochondrial protein-coding genes reconstructed using Bayesian 3.2.0. The best-fit nucleotide substitution model is ‘GTR + G+I’. The support values are shown next to the nodes. Three Triozidae species were included as outgroup taxa. Subfamily-level taxonomy was shown for each taxon.
